# Exploring the effect of problem‐based facilitatory teaching approach on metacognition in nursing education: A quasi‐experimental study of nurse students in Tanzania

**DOI:** 10.1002/nop2.514

**Published:** 2020-07-06

**Authors:** Walter C. Millanzi, Stephen M. Kibusi

**Affiliations:** ^1^ Department of Nursing and Midwifery College of Health Sciences The University of Dodoma Dodoma Tanzania; ^2^ Department of Public Health College of Health Sciences The University of Dodoma Dodoma Tanzania

**Keywords:** curriculum, facilitatory, learning, metacognition, nursing, pedagogy, problem, teaching

## Abstract

**Aim:**

Currently, there has been a progressive shortage of not only the number of frontline healthcare providers but also a decline in the quality of nursing care. There is a growing concern to rethink the approaches on how nurses are prepared, explore and test novel approaches for delivering the nursing curricula. This study tested the effect of the problem‐based facilitatory teaching approach on metacognition among nursing students in Tanzania, higher learning institutions.

**Design:**

A controlled pre‐/post‐test quasi‐experimental study design with a quantitative research approach was employed in this study.

**Methods:**

The study was conducted between February–June 2019 including two purposively selected higher learning institutions in the Dodoma region, the central zone of Tanzania. The 401 randomly selected undergraduate nursing students (interventional = 134 and control = 267) were involved. The auditing inventory developed by the researcher measured the intervention, and the questionnaire titled Metacognition Strategies in Nursing was adopted to measure the metacognition, respectively. Statistical analysis was performed using the Statistical Package for the Social Solution (SPSS) software program version 23.

**Results:**

Findings indicated that 65.8% of the study participants were males. The post‐test findings revealed a significant gain in metacognition scores among participants in an intervention group between (M = 23.27; SD = 1.716) at baseline and (M = 66.31; SD = 6.204) post‐intervention. 63.4% of the total sample in an intervention group demonstrated a high level of knowledge about the regulation of cognition compared to their counterpart control group. However, 69.1% (*N* = 85) participants in the control group performed better for the knowledge about cognition. With the control of other factors, the intervention was found to be more times likely to influence metacognition among nurse students (AOR = 1.603, *p* < 0.05, 95% CI: 1.023, 2.513) . In conclusion, the intervention had the potential to positively effect the levels of metacognition among nurse students. Hence, it was closely linked to professional competency and it would change the spectrum of nursing competency and quality of care among nurse students.

## 1 BACKGROUND

Nursing profession represents a glue that holds patients' experience with the healthcare system. However, there is a critical shortage of nurses despite increasing health needs and demands for an expanded role of the nursing profession in the delivery of complete health care in the globe (WHO, 2016). As of 2006 World Health Organization (WHO) report, it was estimated that countries with fewer than 23 physicians, nurses and midwives per 10,000 inhabitants fail to achieve adequate coverage of quality and cost‐effective healthcare services to the people (Guilbert, 2006). Nursing represents the glue that holds the patient's experience with the healthcare system.

Across the entire spectrum of care needs and patient role experiences, there is a high demand for competent professional care and support from the nurses. Currently, nursing professionals make almost 50% of the health workforce worldwide. Of the 43.5 million healthcare workers, 20.7 million (50%) are nurses and midwives. Global projection of the shortage of nurses and midwives by 2030 is in a moderate decline by 7.6 million for the developed countries, whereas trends show a worsening situation for the African and Eastern Mediterranean regions if the current trend continues (WHO, 2016, p. 6).

According to the Human Resource for Health Strategic Plan 2008–2013 report by Ministry of Health and Social Welfare (2008), the shortage of nurses in Tanzania ranged from 65% in public health facilities and 86% in private health facilities, indicating that Tanzania's health system faces a shortage of nurses, which requires urgent measures. On the other hand, increased burden of new diseases, increased population aged over 60 years old, advances in science and technology, increased patient autonomy and demands for quality and affordable care, all call for new thinking towards nursing as a profession, its body of knowledge, learning pedagogy and scope of practice (Bing‐Jonsson, Hofoss, Kirkevold, Bjørk, & Foss, 2016; WHO, 2016, 2017).

The changes to the healthcare system have significant pedagogical implications in nursing education where experts agree that without a radical change in it, nursing graduates will lack metacognitive skills and be ill‐prepared for practice (Chiejina & Ebenebe, 2013; Hsu & Hsieh, 2014). However, national leaders in health care, nursing and other stakeholders have outlined the need for the transformation of nursing education and recommend innovative pedagogical approaches to improve the current model of preparing nurses for current and future nursing practice environment (Dolamo & Olubiyi, 2013; Souza, Venkatesaperumal, & Radhakrishnan, 2013; WHO, 2013, 2016). This study believed that innovative approaches to preparing nurses in their metacognition would empower them to function effectively and independently in community settings, understand the coordination of care and collaborate with healthcare team members. They would also demonstrate abilities to recognize how policies and regulations affect patients' access to health care and how they have an impact on patients' health outcomes.

Corresponding to the complexity of the nursing body of knowledge and the expanding roles that graduate nurses are expected to carry, today the nursing curriculum is highly loaded with both robust content and rich practical experiences (Birks, Ralph, Cant, Hillman, & Tie, 2015). It appears that nursing curricula are lined up to ensure that nursing graduates have higher levels in metacognition, and are critical thinkers who who can deliver evidence‐based health services to people and thus, become the real‐world problem solvers (Chiejina & Ebenebe, 2013; Ojemeni et al., 2017).

Metacognition is an umbrella term based on brain structures for quality assurance; it denotes an individual's actual mental plan on how to approach a given learning task, monitoring and comprehension, solve problems and evaluate progress towards the completion of a given task in a rapidly changing environment (Hakimzadeh, Ghodrati, Karamdost, Applin et al., 2011). Measuring metacognition remains a critical issue among researchers in education, whereas it is currently acceptable to view this mental phenomenon at two levels: knowledge about cognition (declarative knowledge, procedural knowledge and conditional knowledge) and regulation of cognition (planning, information management strategies, comprehension monitoring, debugging strategies and evaluation) (Applin et al., 2011).

Apart from having an overall critical shortage, only 6.6% of the present teaching staff in developing countries has formal preparation in education (WHO, 2013). There is, therefore, challenges related to the shortage of faculty but perhaps most importantly limited competencies for teaching among most of the educators which as a result makes most of them to predominantly devote to using instructional methods of teaching (Makunja, 2016). Moreover, this is understandable because instructional teaching methods (traditional) are cheaper, easy to implement, can cover an extensive course content at once and suitable for a large group of students (Bishaw & Egiziabher, 2013). Consequently, this has a profound negative effect on the metacognition and overall development of the learners (Paslawski, Kearney, & White, 2014). In this case, students tend to associate learning with the process of merely preparing for a standardized test and/or earning a grade (Makunja, 2016; NACNEP, 2010).

This is completely counterproductive because nursing, unlike other professions, heavily involves both learning by heart and learning by mind. At its heart is about nurturing the passion and devotion to unconditionally care for those in need and respect for human dignity (McAfee & Bell ; Ojemeni et al., 2017). This is supported by the mind, in the form of rigorous core learning of the science and art of nursing. A global strategic direction 2016–2020 is to strengthen nursing and midwifery education. It has begun involving various stakeholders (expertise) and empowering educational systems (WHO, 2016). Emphasis has been directed towards developing and implementing competency‐based curricula particularly in developing countries where the situation is much worse (Komba & Mwandanji, 2015; Makunja, 2016; Paulo, 2014; Marijani, 2017).

Tanzania, through the Ministry for Health, Community Development, Gender Elderly and Children (MoHCDGEC), has introduced the competency‐based curriculum for nursing (Marijani, 2017). However, data show little changes in terms of failure rate and overall poor performance in nursing programmes (Swai, 2017; Gemuhay, Kalolo, Mirisho, Chipwaza, & Nyangena, 2019; Tjoflåt & Våga, 2017). Reports on and about unethical and illegal practices, under standard care and malpractices are not uncommon around the nation. Although nursing education is slow in adapting changes in healthcare systems, a pedagogical shift away from lecture and memorization approaches has been recommended (Jamshidi, Molazem, Sharif, Torabizadeh, & Kalyani, 2016; Stanley & Dougherty, 2010).

Teaching and learning pedagogies that have potential influence in empowering learners with metacognitive and non‐metacognitive skills include problem‐based learning, case study, team‐based learning, simulation with debriefing, reflective writing and portfolio writing (Jost, Brüstle, Giesler, Rijntjes, & Brich, 2017; Lawal, Weaver, Bryan, & Lindo, 2015; Ulrich, Early, Krozek, & Ashlock, 2010). Competency‐based curricula aligned with facilitation in a problem‐based environment (FPBE) has a greater potential to improve learning outcomes in nursing (Paulo, 2014). The impact of problem‐based learning has already been reported in developed countries such as Canada, the United States, Sweden, Holland, England, Japan and Denmark (Burgess, Ayton, & Mellis, 2016; Egelund, Peter, & Jensen, ; Masek & Yamin, 2012).

Contrary to the conventional pedagogy, it has been reported that the problem‐based learning approach exposes learners as early into critical health incidences (real‐world health problems) and clinical practices as possible (Bokey, Chapuis, & Dent, 2014; Masek & Yamin, 2012; Wafaa & Nahed, 2010). The early facilitated, supervised and mentored clinical practices among nurses help to empower them with metacognitive and non‐metacognitive skills and work independently and effectively in this era of a statistically significant shortage of healthcare workers and nurse educators (Celiker, 2015; Tsigarides, Wingfield, & Kulendran, 2017). On the other hand, based on the reviewed literaure, it appears that nursing education is faced by a challenge of the tacit assumption that nurses with clinical expertise and content knowledge are qualified to teach nursing students (Ismail, Aboushady, & Eswi, 2015; Ojemeni et al., 2017; Souza et al., 2013).

However, many master nurse practitioners who are hired in higher training institutions and serve as nurse educators have little or no training in pedagogical principles, which make them focus more on content than improving their teachings (Melnyk, Fineout‐Overholt, Gallagher‐Ford, & Kaplan, 2012; Stanley & Dougherty, 2010). Moreover, with limited pedagogical knowledge and skills, nurse educators tend to produce the teaching and learning environment where they were taught, focusing on delivering content than on students' learning. Limited nurse educator training and faculty development significantly affect the landscape of nursing education that needs to be improved.

The aforementioned changes in the healthcare system and expectation of a new nurse to address the shortage, the limited number of nurse educators, need to incorporate new participatory teaching and learning pedagogies to prepare nurses both to learn and to practise (Brown & Crookes, 2016). It was a belief of this study that the use of problem‐based learning approach promotes learner‐centered teaching and learning activities. Its use in this study would address both, the pedagogical deficits and empowerment of nurse educators with pedagogical skills, which are potential in facilitating learning among nurse students. The pedagogy would give a potential solution to the shortage of both competent nurses and nurse educators. It would also fill the gap in pedagogical emphasis on thinking about metacognitive learning in nursing education.

The integration of problem‐based learning to the nursing curriculum and measuring its effectiveness in improving nursing learning outcomes is not well established in Tanzania and for most countries in the region. It is possible to find various problem‐based methods being used here and there, but this comes usually under the discretion of the educators and not from a well‐guided directive from the curricular (Langtree, 2014). Based on the reviewed literature, it appears that understanding to what extent teaching methods like FPBE pedagogy could be an alternative and effective teaching pedagogy in improving metacognition to nursing students is important (Jackson, 2016) to inform the needed shift in nursing education and practices.

The current study aimed at determining the effect of FPBE teaching pedagogy on metacognition among undergraduate nursing students in Tanzania. The study was believed to be potential as it would provide improvement in faculty pedagogical skills and those empower nurse students with metacognition would make them work independently and effectively in the shortage of nurses. The metacognition was assessed in two levels including knowledge about cognition and the regulation of cognition. The tested research hypothesis stated that there was no statistically significant difference in the levels of metacognition between nurse students under facilitation in a problem‐based environment (FPBE) and their counterparts in the non‐facilitation in a problem‐based environment (NFPBE) in a higher learning institution, Tanzania.

## 2 RESEARCH METHODS AND MATERIALS

### 2.1 Study design and approach

The study was a pre‐/post‐test quasi‐experimental with a controlled study of random allocated learning institutions (to either be in an intervention or control group) through a simple random sampling technique by lottery method, which was done by the research assistants. The researcher blinded the study participants and research assistants about their allocation to either the intervention or the control group before the study. Pieces of paper labelled “institution one and institution two,” were then folded into a box and shacked by the research assistant. Then, the first pick of papers after opening it was assigned to an intervention and the second to the control group. 401 study participants were purposively selected (interventional group = 134 and control group = 267) by the researcher and research assistants.

The participants were undergraduate nurse students from selected government and private‐owned higher learning institutions in Tanzania. This population was among the greatest expected workforce in the nursing profession who could work in various health facilities to render health service among people. If they would graduate competently under proper and excellent academic preparation, it would help to improve the quality of health services to the society at a low cost. The quantitative research approach was employed to determine the extent FPBE could improve levels of motivation to learn among nurse students.

The study included undergraduate nurse students, who were admitted and registered directly from higher education and stay in/out the campus in the respective universities as per the semester schedule. Moreover, nurse students, who were not repeating the year of studies or transferred in from other universities or upgrading, students who had a regular attendance of classes and those who gave a written informed consent (willingness to join the study) before the commencement of the study. Matching of the study participants by their social demographic and academic characteristics was also done to ensure the similarities.

The sample size was determined by using findings of Shahin et al. (2013), who did a study on the critical thinking and self‐directed learning as an outcome of facilitation in a problem‐based environment among nurse students. The study showed that there was a statistically significant difference in mean scores for *SD* 16.44 in an intervention group (A) and *SD* 14.45 in the control group (B). A WinPepi Software program (sample size calculator) version 11.65 was used to calculate the minimum sample of this study were eligible to join it. Effect size (*d* = 4.5) of demonstrating a statistically significant difference between mean values before and after the intervention was set at a 95% confidence interval. A significance level was set at 5% (*p* < 0.05) with the power of 80%. The ratio of sample size was B:A = 1:2.

### 2.2 Study location

The study was done between February–June 2018 in the two major universities within Dodoma administrative region and currently the capital city, central zone of Tanzania. The pre‐/post‐written test was used to collect and compare data before and after an intervention. The interventional group learned the prepared research content by using FPBE pedagogy, whereas their counterpart (control group) learned by using the conventional‐based instructional method as non‐facilitation in a problem‐based environment teaching approach.

### 2.3 Data collection process

The research instruments were administered to the study participants by the researcher and assistant researchers to facilitate the work and ensure accurate information was collected from them. All participants answered the same questions before and after the intervention. No kind of harm (be physical, emotional, social, spiritual, cultural or economic) occurred to the study participants throughout the study. Before the commencement of data collection, written informed consents were obtained from the study respondents that helped the research to be assured about their willingness to participate in this study. To ensure privacy and confidentiality, all the study participants gathered in a room that was offered by the deans of the respective learning institution, and were given brief instructions on how to fill the questionnaires. Then, the researcher and research assistants distributed copies of questionnaires among the study participants and supervise the process of filling them throughout data collection. For any point that needed clarifications, the researcher or assistants responded accordingly. Anonymity was ensured by excluding the study participants' names from the data collection instruments.

### 2.4 Data collection tools

The instrument used for data collection was a structured questionnaire titled Metacognition Strategies in Nursing (QMCSN), which had two parts. It was adapted to measure the levels of metacognition among undergraduate nurse students. Part A of the instrument had nine (9) items that elicited information about demographic data (e.g. age, sex, education level of the student and accommodation status). Part B elicited information about levels of metacognition adopted by students in their learning processes. This part had fifty‐two (52) items, which covered two domains (knowledge about cognition, *N* = 23 items, and regulation of cognition, *N* = 29 items). The questionnaire items in part B adopted a two‐point ordinal scale ranging from 0 (false) to 1 (true). The “false” response indicated that the study participant did not possess the action explained by the item, whereas the “true” responses indicated that they possessed.

The items under the aspect of knowledge about cognition meant to measure the low level of metacognition, whereby the participant who scored <11.5 was considered having a low level of knowledge about cognition. Metacognition in the domain of the regulation of cognition measured the higher level of the study participants to comprehend what was meant by them to learn. Study participants who scored >14.5 were considered to have a high level of regulation of cognition, otherwise not. The two domains of metacognition were then analytically transformed, and the new variable was computed to determine the overall metacognition among the study participants. Participants who scored >21 of the metacognition scores (52 scores) were considered having a high level of metacognition.

### 2.5 Validity

This study used the content validation to validate the structured questionnaires that intended to measure the levels of metacognition among the study participants and the auditing inventory developed by the researcher to measure the intervention. The researcher developed the research tools by benchmarking research tools from previous studies before being shared with the supervisor and subject matter for professional assistance including inputs, deletion and correction. The comments from the supervisory team included addressing grammatical errors found in the tools and rewriting subheadings in a clear and concise language at the level of the study participants. No item was either added or deleted. The researcher of this study addressed the comments accordingly, and the revised tools were then shared again with the supervisory team for them to re‐cross‐check them, whereas no additional comments were given.

### 2.6 Reliability

The tool was tested for the content, language accuracy, clearness and ability of the study respondents to understand the content to assure the reliability of the information, which would be provided by the study participants. It was pre‐tested by the researcher with professional support from supervisors, subject matter and statisticians from the University of Dodoma before the actual field use. A pilot study involving 20 consented respondents was conducted at Bugando School of Nursing, which was a location other than the sampled study area to test the abilities of the tool for it to give the intended results. The 20 copies of questionnaires were distributed to the sampled study respondents during the pilot study after having them seated on chairs in the room and given a brief instruction on how to fill them. None of the items were detected to be difficult or ambiguous among the participants, and therefore, they were all retained. However, grammatical corrections were done accordingly.

Findings from the pilot study were subjected to the scale analysis by using a Statistical Package for the Social Solution (SPSS) software program version 23. No item weighed less, and thus, none of them was extracted from the scale. The results of the scale analysis revealed a Cronbach *α* of .71, which was statistically accepted as an indicator that the tool was reliable to be used in this study for the field data collection.

## 3 DEVELOPMENT AND CLASSROOM TRYOUTS OF THE RESEARCH TEACHING GUIDELINES

Auditing inventory (AI), developed by the researcher, was used to collect experts' and students' opinions of the developed research teaching materials after each classroom tryouts before the actual field testing. The developed material was tried out in the classroom for three phases (phases 0, 1 and 2) (Mafumiko, 2008). Phase 0 was the development phase, while phases 1 and 2 were for classroom tryouts. The third version was subjected to the field testing. Classroom tryouts for phases 1 and 2 were done in one sampled health training institution which was different from health training institutions where the study was conducted. All classroom tryouts involved 10 nurse students, researcher, 2 research assistants, 1 curriculum development expert and 1 nurse tutor with over 5 years of experience in teaching leadership and management content.

Experts' and student's opinions of one classroom tryout led to the development and refinement of the next version. Auditing inventory covered several aspects including the relevance of the content, content organization, organization of learning experiences, timing, dosage, frequency and evaluation strategies. The topics were drawn from the leadership and management course, which was found in the undergraduate curriculum for Bachelor Degrees in Nursing Program. The researcher developed the ill‐structured scenario on conflict resolution strategies at a working area with assistance from the curriculum development expert and nurse tutor who had over 5 years of teaching experience in nursing courses.

The evaluation process of the developed research teaching material was done formative and summative based on the experts' and students' opinions. All observations from experts and students were only used to assure the acceptability, feasibility and practicality of the research teaching and learning material before actual field implementation.

### 3.1 Validation and reliability of the research instruments

All research tools were shared with experts and senior colleagues for their review, inputs and opinions to ascertain their validity before classroom tryouts (done in phase 0). The recommended inputs from the experts included: to organize the content from simple to complex, the inclusion of video‐based scenarios that would reflect the real‐world leadership and management situations encountered by nurses in the clinical setting and allocating the time (minutes) based on the dosage of the content per session. Amendments of the content, dosage, organization, timing, teaching and learning activities and the evaluator approach of the learning process comments from experts were addressed accordingly to ensure that only the recommended and advised content and items were administered to the sampled students.

### 3.2 Facilitation in a problem‐based environment sessions (treatment)

This part served as an actual implementation phase, which involved the followings:

#### 3.2.1 Introduction and group formulation

This part was covered in the first day of the study for introducing the FPBE and familiarizes students with the researcher and research assistants. The researcher introduced the FPBE process and shared the expected terminal behaviour throughout the FPBE classes. Students were then randomly assigned to the learning groups (eight students per each group) whereby they were asked to appoint leaders and record keepers among themselves. This part took approximately 30 min.

#### 3.2.2 Problem presentation, solving and discussions

The researcher and research assistant before its commencement to avoid groups going off track reviewed objective of each session. Each group was asked to seat in the round so that they could maintain eye contact and facilitate the easy flow of discussions. Thereafter, each group was given the developed scenario on conflict resolution at the working place and allows the students to start addressing it. Participants were guided and facilitated by the researcher and research assistants to solve problems, listing what they knew, what they did not know, what they needed to know, plan, implement and evaluate their learning activities.

Students were guided to clarify, rank and assign learning tasks to each member of the group. They were then guided to identify and suggest the reasoned available resources needed to solve the presented problem and continue solving it. This part took approximately 60–120 min based on the institution schedules. Then, students were given 1 week to address the problem until the next scheduled time. The purpose was to enable them identify learning iisues to be explored about the problem. As part of the closure, the researcher and research assistants required either students to communicate by mobile texts, orally or by writings through email whenever they need any help or clarifications. In the next meeting, each group presentated startegies that would be used to solve problems, which were assigned among them. Presented tasks were followed by discussions and sharing of real‐world ideas, problems, which reflected their experiences in real life. Misconceptions and other myths were then cleared, and participants gained new knowledge and skills about how to address conflict in working areas once they occur.

#### 3.2.3 Group facilitations

This study used two forms of facilitating the groups including researcher and research assistant facilitation and group leaders' facilitation. Group leaders were sometimes used to facilitate groups because the classes were so large. They were briefly instructed to act as facilitators on how to monitor and control the learning process in their groups. They were given the roles of moving from one group to another, ask probing questions and give encouraging words, which in turn could help to serve students who would nearly drop or withdraw from the study.

#### 3.2.4 Assessments

After completing the FPBE classroom sessions, the researcher, assistant researchers and students evaluated the lesson objectives by providing inputs, students learning behaviours, advantages and disadvantages of group interactions and the benefits of learning through FPBE. Peer assessment was the main method used to assess the learning process among participants. The post‐test to assess end line levels of metacognition was then administered to the participated students to ascertain the effect of FPBE.

#### 3.2.5 Data analysis

Descriptive and inferential statistical analyses were performed in this study. All the statistical analysis was performed using the Statistical Package for the Social Solutions (SPSS) version 23. The study findings are presented in tables. Descriptive statistics by the means of chi‐squared and cross‐tabulation statistical tests were performed to determine the relationship between categorical variables, and findings were presented in frequencies, percentages, mean scores and standard deviation (*SD*). Paired‐sample and independent‐samples *t* tests were performed to compare the differences of the mean scores among the study participants within and between groups, whereby mean (*M*), standard deviation (*SD*) and *p*‐value were used to present the findings in tables. Inferential statistical analyses were performed through regression analysis to determine the association between variables. The findings were presented in tables by odds ratio (OR), adjusted odds ratio (AOR) and *p*‐value that was set at ≤0.05 to be statistically significant at 95% confidence interval (CI).

## 4 RESULTS

### 4.1 Demographic characteristics of the study participants

Table 1 and show the descriptive statistics of the measured variables. Findings revealed that 65.8% of the study participants were males and 34.2% females. Many participants (73.6%) had age ranging between 25–29 years with (*p* > 0.05) of their gender and age distributions between groups. It was observed that 92.5% of participants were singles and 69.3% of them lived on campus. Moreover, 73.8% and 75.3% of participants were interested and satisfied in pursuing the nursing programme and its courses, respectively. However, 30.7% and 13,7% participants experienced trouble in comprehending course contents due to its complexity (that the contents were difficult for them to gasp meanings) and inadequate support from instructors of which the experiences varied among participants between groups. A 19.9% of the participants experienced difficulties in accessing updated learning materials while 0.10% participants faced unconducive environment in favor to their learning processes. Other findings were found as shown in the table.

**TABLE 1:** Distributions of participants' sex, age, marital status and accommodation status between FPBE and NFPBE group (*N* = 401)

Variable	FPBE	NFPBE	*p*‐value
	*n*(%)	*n*(%)	
Gender			
Males	83(61.9)	181(67.8)	0.244
Females	51(38.1)	86(32.2)	
Age			
<24 years	6(4.5)	25(9.4)	0.192
25–29 years	100(74.6)	195(73.0)	
> 30 years	28(20.9)	47(17.6)	
Marital status			
Single	123**(**91.8)	248**(**92.9)	0.695
Married	11(8.2)	19(7.1)	
In campus			
Yes	43**(**32.1)	235**(**88.0)	0.001
No	91(67.9)	32(12.0)	

**TABLE 2:** Mean scores difference of metacognition among the study participants between groups (*N* = 401)

Metacognition			
Variables	Pretest	Posttest	*p*‐value
	M (*SD*)	M (*SD*)	
FPBE	23.27 (1.716)	66.31 (6.204)	0.001
NFPBE	22.73 (1.302)	45.71 (3.621)	

### 4.2 Mean scores difference of metacognition among the study participants between groups

Mean scores difference of metacognition among nurse students was determined between baseline and posttest. Findings in Table 3 indicate that there was no statistically significant difference in baseline mean scores of metacognition in an intervention (M = 23.27; SD = 1.716) compared to a control group (M = 22.73; 1.302). However, a significant gain of the overall mean scores of metacognition between groups was observed at posttest whereby participants in an intervention group scored higher (M = 66.31; SD = 6.204) than those in the control (M = 45.71; SD = 3.621) (P<0.01).

**TABLE 3:** Distributions of participants' interests, reasons for choosing nurse as a career, satisfaction in the nursing profession and its programmes, benefits and learning difficulties between FPBE and NFPBE group

Variable	FPBE	NFPBE	Chi‐squared test		
	*n*(%)	*n*(%)	Value	*df*	*p*‐value
Interest					
Yes	92(68.7)	204(76.4)	2.771	1	0.096
No	42(31.3)	63(23.6)			
Reasons to choose nurse					
Own choice	71**(**53.0)	139**(**52.1)	0.430	3	0.934
Parent's/peer's pressure	29(21.6)	55(20.6)			
Easier to get a job	24(17.9)	48(18.0)			
Entry qualifications	10(7.5)	25(9.4)			
Satisfaction					
Yes	78**(**58.2)	224**(**83.9)	31.660	1	0.001
No	56(41.8)	43(16.1)			
Learning benefits					
Agreed	104**(**77.6)	233**(**87.3)	6.200	1	0.013
Disagreed	30(22.4)	34(12.7)	6.200		
Learning difficulties					
Difficult accessing updated learning materials	24**(**17.9)	56**(**21.0)	9.665	4	0.046
Complex course contents	49(36.6)	74(27.7)			
Inadequate support from lecturers	18(13.4)	37(13.9)			
Limited time	25(18.7)	79(29.6)			
No conducive environment	18(13.4)	21(7.9)			

### 4.3 The mean score differences of the domains of metacognition among the study participants between groups

The metacognition in this study was further measured quantitatively into two domains including the knowledge about cognition and knowledge about the regulation of cognition, respectively (Tables 4, 5, 6, 7, 8)

**TABLE 4:** The post‐test levels of metacognition among the study participants (*N* = 401)

Variables	Yes	No	*X^2^*
	*n*(%)	*n*(%)	*p*‐value
Intervention			
FPBE	112(83.6)	22(16.4)	5.969
NFPBE	123(46.1)	144(53.9)	0.017
Total	235(58.6)	166(41.4)	

Note

The post‐test levels of metacognition among the study participants: Table4  indicates that out of 401 study participants, 58.6% (*N* = 235) had a higher level of metacognition. However, 83.6% (*N* = 112) of the study participants in an intervention group had adequate metacognition against their counterparts in the control group (46.1%; *N* = 123). The difference in metacognition levels among the study participants was observed to be statistically significant (*p* < 0.05).

**TABLE 5:** Distributions of the level of knowledge about cognition and regulation of cognition, among participants between FPBE (*N* = 112) and NFPBE (*N* = 123), respectively

Variables	Knowledge about cognition		Regulation of cognition	
	Yes(%)	No(%)	Yes(%)	No(%)
FPBE	41(36.6)	71(63.4)	71(63.4)	41(36.6)
NFPBE	85(69.1)	38(21.9)	38(21.9)	85(69.1)
Total	126(53.1)	109(46.9)	109(46.9)	126(53.1)

Note

The levels of domains of metacognition among the study participants between groups: Table5  shows that out of 235 study participants who demonstrated a high level of metacognition, 37.9% (*N* = 109) of them had a higher level of knowledge about the regulation of cognition against 53.1% (*N* = 126) participants who demonstrated high levels of knowledge about cognition. However, 63.4% (*N* = 71) out of 109 study participants who demonstrated high levels of regulation of cognition were from the intervention group. Moreover, findings revealed that out of 126 participants who demonstrated high levels of knowledge about cognition, 69.1% (*N* = 85) were from the control group.

### 4.4 Knowledge about cognition scores

This domain was measured in three aspects including declarative, procedural and conditional knowledge. As shown in Table 6 , the baseline declarative knowledge scores among the study participants did not differ significantly as in an intervention group was (*N* = 134, mean = 55.35, *SD* = 12.68) and the control group (*N* = 267, mean = 57.80, *SD* = 12.39, *t* (399) = −1.856, *p* > 0.05, 95% CI: −5.0511, 0.1451). However, statistically significant difference was observed post‐test whereby participants in an intervention group scored low (*N* = 134, mean = 64.76, *SD* = 12.47) than the control group (*N* = 267, mean = 67.57, *SD* = 9.47, *t* (399) = −2.508, *p* < 0.01, 95% CI: −5.005, −0.607).

**TABLE 6:** Mean score differences of knowledge about cognition in the domains of declarative, procedural and conditional knowledge mean scores, among participants between FPBE (*N* = 134) and NFPBE (*N* = 267)

Pre‐intervention						Post‐intervention				
Variables	FPBE	NFPBE	Independent *t* test			FPBE	NFPBE	Independent *t* test		
	*M*(*SD*)	*M*(*SD*)	*p*‐value	95% CI		*M*(*SD*)	*M*(*SD*)	*p*‐value	95% CI	
				Lower	Upper				Lower	Upper
Declarative knowledge	55.35(12.68)	57.80(12.39)	0.064	−5.051	0.145	64.76(12.47)	67.57(9.47)	0.013	−5.005	−0.607
Procedural knowledge	56.68(12.52)	57.12(11.94)	0.058	−4.967	0.084	61.44(16.21)	66.01(12.15)	0.002	−7.414	−1.736
Conditional knowledge	51.94(10.76)	54.09(10.55)	0.057	−4.359	0.061	62.06(14.35)	65.76(11.00)	0.005	−6.239	−1.152

Regarding the baseline procedural knowledge, participants' scores between groups did not differ significantly between groups at baseline, whereas participants in an intervention group scored (*N* = 134, mean = 54.68, *SD* = 12.52) and the control group (*N* = 267, mean = 57.12, *SD* = 11.94, *t* (399) = −1.900, *p* > 0.05, 95%CI: −4.967, 0.084). Statistically significant difference in mean scores between groups was observed with the post‐test mean scores where participants in an intervention group scored low (*N* = 134, mean = 61.44, *SD* = 16.21) than participants in the control group (*N* = 267, mean = 66.01, *SD* = 12.15, *t* (399) = −3.168, *p* < 0.01, 95% CI: −7.414, −1.736) (Table 6).

Baseline conditional knowledge scores among study participants between groups did not differ significantly as findings have shown the scores in an interventional group were approximately the same (*N* = 134, mean = 51.94, *SD* = 10.76) as those of the control group (*N* = 267, mean = 54.09, *SD* = 10.55, *t* (399) = −1.912, *p* > 0.05, 95% CI: −4.359, 0.061). The baseline findings were unlike with the post‐test scores where participants in an intervention group scored low (*N* = 134, mean = 62.06, *SD* = 14.35) than participants in the control group (*N* = 267, mean = 65.76, *SD* = 11.00, *t* (399) = −2.856, *p* < 0.01, 85% CI: −6.239, −1.152) (Table 6).

### 4.5 The overall mean score differences of participants' knowledge about regulation of cognition between groups

As shown in Table 7, study participants in an intervention group scored higher in the aspect of regulation of cognition (*N* = 134, mean = 1.52, *SD* = 0.501) than participants in the control group (*N* = 267, mean = 1.40, *SD* = 0.490, *t* (399) = 2.398, *p* < 0.05, 95% CI: 0.023, 0.228).

**TABLE 7:** Mean score differences of participants' knowledge about regulation of cognition between groups (*N* = 401)

Variables	*N*	M	*SD*	*df*	CI 95%		*t*‐value	*p*‐value
					Lower	Upper		
FPBE	134	1.52	0.501					
NFPBE	267	1.40	0.490	399	0.023	0.228	2.398	0.017

Note

The overall mean score differences of participants' knowledge about regulation of cognition between groups: As shown in Table7 , study participants in an intervention group scored higher in the aspect of regulation of cognition (*N* = 134, mean = 1.52, *SD* = 0.501) than participants in the control group (*N* = 267, mean = 1.40, *SD* = 0.490, *t* (399) = 2.398, *p* < 0.05,95% CI: 0.023, 0.228).

### 4.6 The mean score differences of the domains of knowledge about regulation of cognition among the study participants between groups

The aspect was quantitatively measured in five aspects including planning (P), information management strategies (IMS), comprehension monitoring (CM), debugging strategies (DS) and evaluation knowledge (EK). It was observed that there was no statistically significant difference between the baseline planning knowledge scores of an interventional group (*N* = 134, mean = 52.84, *SD* = 11.79) and the control group (*N* = 267, mean = 54.99, *SD* = 10.33, *t* (399) = −1.868, *p* > 0.05, 95%CI: −4.399, 0.113). However, postintervention mean scores differed significantly from the baseline whereas participants in an intervention group scored higher (*N* = 134, mean = 62.98, *SD* = 13.70) than participants in the control group (*N* = 267, mean = 55.50, *SD* = 11.49, *t* (399) = 5.788, *p* < 0.01, 95%CI: −4.939, 10.021).

**TABLE 8:** Mean score differences of regulation of cognition in the aspects of planning (P), information management strategies (IMS), comprehension monitoring (CM), debugging strategies (DS) and evaluation knowledge (EK) mean scores, among participants between FPBE (*N* = 134) and NFPBE (*N* = 267)

Pre‐intervention						Post‐intervention				
Variables	FPBE	NFPBE	Independent *t* test			FPBE	NFPBE	Independent *t* test		
	*M*(*SD*)	*M*(*SD*)	*p*‐value	95% CI		*M*(*SD*)	*M*(*SD*)	*p*‐value	95% CI	
				Lower	Upper				Lower	Upper
P	52.84(11.79)	54.99(10.33)	0.068	−4.399	0.113	62.98(13.70)	55.50(11.39)	0.001	4.939	10.021
IMS	51.12(11.64)	54.96(10.40)	0.060	−0.092	4.417	69.70(12.70)	53.06(12.49)	0.001	14.021	19.250
CM	60.16(13.01)	58.19(8.78)	0.073	−0.187	4.135	64.42(13.59)	55.49(10.82)	0.001	6.477	11.397
DS	55.11(10.86)	53.10(10.21)	0.069	−0.161	4.182	57.20(12.04)	53.21(11.02)	0.001	1.629	6.360
EK	53.97(10.97)	51.99(9.69)	0.066	−0.129	4.091	55.77(11.21)	52.36(11.88)	0.006	0.983	5.838

The aspect of how participants had the information management strategies knowledge was also assessed. Findings revealed that the mean score among participants between groups did not differ significantly when compared to an interventional group (*N* = 134, mean = 51.12, *SD* = 11.64) and the control group (*N* = 267, mean = 54.96, *SD* = 10.40, *t* (399) = 1.886, *p* > 0.05, 95% CI: −0.092, 4.417). After an intervention, participants in an intervention group scored higher (*N* = 134, mean = 69.70, *SD* = 12.70) than the control group (*N* = 267, mean = 53.06, *SD* = 12.49, *t* (399) = 12.509, *p* < 0.01, 95%CI: 14.021, 19.250).

There was no observed statistically significant difference in mean scores of comprehension monitoring knowledge among the study participants where participants in an intervention group scored approximately the same (*N* = 134, mean = 60.16, *SD* = 13.01) as the control group (*N* = 267, mean = 58.19, *SD* = 8.78, *t* (399) = 1.796, *p* > 0.05, 95%CI: −0.187, 4.135). The statistically significant difference in mean score was revealed in the postinterventional findings whereby participants in an intervention group scored high (*N* = 134, mean = 64.42, *SD* = 13.59) than the control group (*N* = 267, mean = 55.49, *SD* = 10.82, *t* (399) = 7.143, *p* < 0.01, 95% CI: 6.477, 11.397).

Nevertheless, the baseline debugging strategies knowledge scores among study participants between groups were not statistically different as those in an interventional group scored nearly the same (*N* = 134, mean = 55.11, *SD* = 10.86) as the control group (*N* = 267, mean = 53.10, *SD* = 10.21, *t* (399) = 1.820, *p* > 0.05, 95%CI: −0.161, 4.182). The performance was statistically different after an intervention whereby the participants in an intervention group scored high (*N* = 134, mean = 57.20, *SD* = 12.04) than their counterparts in the control group (*N* = 267, mean = 53.21, *SD* = 11.02, *t* (399) = 3.319, *p* < 0.01, 95%CI: 1.629, 6.360).

Despite that, Table 8 also expresses the learning evaluation in an interventional group (*N* = 134, mean = 53.97, *SD* = 10.97) and the control group (*N* = 267, mean = 51.99, *SD* = 9.69, *t* (399) = 1.846, *p* > 0.05, 95% CI: −0.129, 4.091). However, postintervention findings in an intervention group were (*N* = 134, mean = 55.77, *SD* = 11.21) and the control group (*N* = 267, mean = 52.36, *SD* = 11.88, *t* (399) = 2.762, *p* < 0.01, 95% CI: 0.983, 5.838).

### 4.7 Factors related to the effect of FPBE teaching pedagogy on metacognition, among the study participants

One of the major aims of the current study was to investigate the effect of FPBE in conjunction with other factors, which seemed to be related to the development of metacognition among the study participants. As shown in Table 9 below, some of the factors which showed statistically significant relationship with metacognition were the intervention (FPBE) (*X* ^2^ = 5.969^a^, *p* < 0.05), gender (*X* ^2^ = 17.776^a^, *p* < 0.01), interest in nursing programmes (*X* ^2^ = 3.424^a^, *p* < 0.05), satisfaction in nursing courses (*X* ^2^ = 3.980^a^, *p* < 0.05) and learning difficulties (*X* ^2^ = 24.457^a^, *p* < 0.01). However, other factors were not related to the levels of metacognition.

**TABLE 9:** (a) Factors related to the levels of metacognition, among study participants (*N* = 401). (b) Effect of FPBE on metacognition among participants between FPBE and NFPBE groups (*N* = 401)

(a)					
Variables	Yes		No		*X^2^*
	*n*(%)		*n*(%)		*p*‐value
Intervention					
FPBE	64(28.4)		70(39.8)		5.969
NFPBE	161(71.6)		106(60.2)		0.017
Gender					
Males	178(74.7)		96(54.5)		17.776
Females	57(25.3)		80(45.5)		0.001
Age					
<24 Yrs.	16(7.1)		15(8.5)		4.235
25 – 30 Yrs.	159(70.7)		136(77.3)		0.120
>30 Yrs.	50(22.2)		25(14.2)		
Marital status					
Singles	206(91.6)		165(93.8)		0.687
Married	19(8.4)		11(6.2)		0.407
Accommodation status					
In campus	158(70.2)		120(68.2)		0.1936
Out campus	67(29.8)		56(31.8)		0.660
Interest					
Yes	158(70.2)		138(78.4)		3.424
No	67(29.8)		38(21.6)		0.044
Satisfaction					
Yes	178(79.1)		124(70.5)		3.980
No	47(20.9)		52(29.5)		0.046
Reasons for choosing nurse as a career					
Own choice	113(50.2)		97(55.1)		
Parents'/peer's pressure	47(20.9)		37(21.0)		1.398
Easier to get a job	44(19.6)		28(15.9)		0.706
Entry qualifications	21(9.3)		14(8.0)		
Learning difficulties					
Inadequate and difficulty in accessing updated learning materials	56(24.9)		24(13.6)		
Complex course contents	58(25.8)		65(36.9)		23.457
Inadequate support from lecturers	36(16.0)		19(10.8)		0.107
Limited time	46(20.4)		58(33.)		
No conducive environment	29(12.9)		10(5.7)		

(b)								
Variables	OR	95% CI		*p*‐value	AOR	95% CI		*p*‐value
		Low	Upper			Low	Upper	
Intervention								
FPBE	1.661	1.093	2.524	0.017	1.603	1.023	2.513	0.04
NFPBE (Ref.)	0.658	—	—	0.001	0	—	—	
Sex								
Males	0.407	0.267	0.621	0	0.424	0.276	0.653	0
Females (Ref.)	1.404	—	—	0.051	0	—	—	
Interest								
Yes	1.45	0.973	2.437	0.065	1.619	1.003	2.611	0.048
No (Ref.)	0.567	—	—	0.005	0	—	—	
Satisfaction								
Yes	1.588	1.006	2.506	0.047	1.214	0.743	1.984	0.439
No (Ref.)	0.697	—	—	0.002	0.518	—	—	
Learning difficulties								
Updated learning materials	0.805	0.339	1.908	0.622	0.867	0.358	2.096	0.751
Complex course contents								
Inadequate support from lecturers	0.308	0.138	0.686	0.004	0.411	0.18	0.939	0.035
Limited time	0.653	0.263	1.621	0.359	0.785	0.307	2.005	0.612
No conducive environment (Ref.)	0.273	0.121	0.619	0.002	0.341	0.148	0.786	0.012
	0	—	—	—	—		—	—

### 4.8 Effect of FPBE and other factors, on metacognition among the study participants between groups

Univariate, binary and multinomial logistic regressions were employed to determine the association between variables and nurse students' metacognition. Table 9 indicates that FPBE was one times more likely to influence the levels of metacognition among study participants (AOR = 1.603, *p* < 0.05, 95% CI: 1.023, 2.513) when adjusted for other factors.

Other demographic characteristics of the participants were also studied. The male gender was observed to be less times likely to positively influence the levels of metacognition than the female gender could do (AOR = 0.424, *p* < 0.01, 95% CI: 0.276, 0.653). Moreover, study participants who were interested in the nursing programme and its course programmes were one time more likely to develop levels of metacognition (AOR = 1.619, *p* < 0.05, 95% CI: 1.003, 2.611). Yet, undergraduate nurse students who experience trouble in their learning process and had no enough time for learning were less times likely to develop levels of metacognition against the study participants who did not experience either of them (AOR = 0.411, *p* < 0.05, 95% CI: 0.180, 0.939) and (AOR = 0.341, *p* < 0.05, 95% CI: 0.148, 0.786), respectively.

## 5 DISCUSSIONS OF THE STUDY FINDINGS

### 5.1 Metacognition among participants

Findings suggest that FPBE teaching pedagogy had a positive effect on undergraduate nurse students' learning outcomes when they were exposed to it. Despite the influence of other factors on the levels of metacognition, it was observed that FPBE could influence the higher level of metacognition (regulation of cognition) than conventional teaching methods could do. The participants' level of metacognition in an intervention group could highly improve from knowledge about cognition to the regulation of cognition among undergraduate nurse students.

The regulation of cognition was an essential aspect of metacognition in this study. The improvement was revealed in the mean score differences on the aspects of the post‐test regulation of cognition including planning, information management and comprehension monitoring, debugging strategies and learning evaluation. Study participants in an intervention group demonstrated the abilities to apply them in their learning process against their counterparts in the control group. They demonstrated highly improved abilities on how to manage time, set learning goals and allocate resources for their learning processes (Planning). Moreover, participants in an intervention group demonstrated improved rates, skills and strategies of organizing, elaborating, focusing, summarizing and evaluating the new important information (information management) they encounter in their learning process than the control group.

However, participants who were subjected to conventional teaching approaches demonstrated abilities to process and use critical thinking skills concerning what was important in their learning process (declarative knowledge). Additionally, they were able to apply their knowledge to implement learning procedures (procedural knowledge) and they knew when to apply the learning process in various situations through discovery, cooperative learning and problem‐solving (conditional knowledge).

There was an improvement in the way participants, particularly the control group, determined under what circumstances specific learning processes or skills could be transferred. On the other hand, study participants in the control group were able to know when and why they could use learning procedures to deliver quality and costeffective health services among patients or clients when they would be at clinical settings. These changes were discussed by the researcher to be statistically significant and were deemed to be due to the effect of the post‐non‐facilitation in a problem‐based environment (NFPBE) teaching pedagogy. Findings above revealed that FPBE teaching pedagogy was more effective on metacognition especially on the part of the regulation of cognition, compared with NFPBE teaching pedagogy, which favoured the development of knowledge about cognition as the lowest level of metacognition.

The observed findings in this study are consistent with some previous studies done elsewhere. For example, findings that were observed by Akpan & Beard (2016) showed problem‐based learning to be effective on academic outcomes, compared with lecture‐based learning. Knowledge becomes outdated very quickly in this knowledge explosion era. Thus, the preparation of nurse students to understand how to learn in a problem‐based environment is essential than equipping students with what to learn. The possession of knowledge is of no use; rather one must ponder over it and use it effectively and appropriately.

Moreover, findings of the current study link with those found by Chiejina and Ebenebe (2013) and Hamdan, Kwan, Khan, Ghafar, and Sihes (2014) who asserted that students' metacognition and problem‐solving skills could more likely be improved when nurse students were exposed to problem‐based learning with the adequate time of learning. The researcher was therefore confident; to note that if well prepared and switched to the environment and available resources, FPBE teaching pedagogy could positively improve nurse students' metacognition higher than conventional teaching pedagogies.

## 6 CONCLUSION AND RECOMMENDATION

This study builds on and extends the earlier research on the effects of FPBE. This was based on the constructivist psychological theory, social constructivism and collaborative learning among a group of undergraduate nurse students regarding the development of metacognition. The findings do throw light on how FPBE effects metacognition. The researcher has a strong belief that nursing students in Tanzania without metacognition in terms of analytical reasoning, real problem‐solving and critical thinking cannot meet the objective of high‐quality patient care and health care reform will be difficult to succeed.

Professional nurses need to have a sharp sense of how to make decisions with regard to what they did evaluate and decide on the validity of their actions related to patient care (Akpan & Beard, 2016). They need to challenge previous assumptions and expand their frame of metacognition and professional practices. Nevertheless, they must realize that their profession is an art of nursing based on scientific knowledge and a high level of reasoning; thus, they are required to justify their actions with evidence that is continuously being collected and assimilated objectively or subjectively.

On the other hand, nursing education is the key to the development of excellence in nursing practices (Heid, 2016). Thus, the move to adopt facilitation in a problem‐based environment, group learning and inquiry learning in nursing education in Tanzania, is worth considering and needs to be continued. The elements of the FPBE, which were used in the current study such as metacognitive learning, collaborative learning, analysis, synthesis and learning in the context of the real problem, have the potential contributions to the development of competent graduate nurses.

Findings from this study suggest that the FPBE strategy is useful and feasible on metacognition, among nursing students in Tanzania. This is a positive and statistically significant finding of the study. The quantitative findings of the current study cannot offer any obvious and strong evidence of sustained development of metacognition through NFPBE. The success of FPBE was attributed to the provision of an opportunity for student's interactions and the fact that students were able to view the learning pathway in the metacognition. This was done through the vantage point of problem identification; they were able to propose learning issues, practise knowledge research and sharing and then revisit the scenario to solve particular real problems.

Therefore, it would be appropriate to reiterate that the researcher is confident in FPBE and believes that it can offer a promising direction to accomplish the goals of developing metacognition to nurse students in Tanzania. A call is made from these findings to all health training institutions to adopt and stipulate FPBE teaching and learning pedagogy into their curricula for the betterment of new graduates who will, in turn, promote the well‐being of the community. Teachers should assist nurse students by making them aware of multiple learning strategies available to them and direct how to recognize an alternative strategy when one is not working.

### 6.1 Limitation of the study

During the implementation phase of this study, group leaders were trained to act as facilitators. This would affect their full participation in solving the presented problems and even make their colleagues not to take into serious their learning roles.

## **AUTHOR** **CONTRIBUTIONS**

W.M: Designed and developed the proposal, exemplary research teaching materials and conducted study interventions. S.M.K.: Shaped of the research idea/concept, reviewed, appraised research teaching materials and research tools, and edited the work.

## ETHICAL APPROVAL

Research Ethics Committee approval and consent to participate: applicable, approved by the University of Dodoma (UDOM) Institutional Research Review Committee (IRRC). Ethics clearance to reach higher training institutions: approved by principals and deans of the respective institutions/schools.

## INFORMED CONSENT

All participants in the current study will be asked for informed consent for their participation.

## References

[nop2514-bib-0001] Akpan, J. P. , & Beard, L. A. (2016). Using constructivist teaching strategies to enhance academic outcomes of students with special needs. Universal Journal of Educational Research, 4, 392–398. 10.13189/ujer.2016.040211

[nop2514-bib-0040] Applin, H. , Williams, B. , Day, R. , & Buro, K (2011). A comparison of competencies between problem‐based learning and non‐problem‐based graduate nurses. Nurse Education Today, 31(2), 129–134. 10.1016/j.nedt.2010.05.003 20817332

[nop2514-bib-0002] Bing‐Jonsson, P. C. , Hofoss, D. , Kirkevold, M. , Bjørk, I. T. , & Foss, C. (2016). Sufficient competence in community elderly care? Results from a competence measurement of nursing staff. BMC Nursing, 15, 1–11. 10.1186/s12912-016-0124-z 26778919 PMC4714519

[nop2514-bib-0003] Birks, M. , Ralph, N. , Cant, R. , Hillman, E. , & Tie, Y. C. (2015). Teaching science content in nursing programs in Australia: A cross‐sectional survey of academics, BMC Nursing, 14, 1–9. 10.1186/s12912-015-0074-x 25995710 PMC4438582

[nop2514-bib-0041] Bishaw, A. E. , Yosef, G (2013). Comparison of Traditional and Constructivist Teaching Approaches in Using English Language Learning Strategies; ( Grade Eleven Students of Bahir Dar Preparatory School ). Ethiop. J. Educ. & Sc., 9(1), 1–13. https://www.ajol.info/index.php/ejesc/article/view/104968.

[nop2514-bib-0004] Bokey, L. , Chapuis, P. H. , & Dent, O. F. (2014). Problem‐based learning in medical education: One of many learning paradigms. Medical Journal of Australia, 201, 134–136. 10.5694/mja13.00060 25128943

[nop2514-bib-0005] Brown, R. A. , & Crookes, P. A. (2016). What level of competency do experienced nurses expect from a newly graduated registered nurse? Results of an Australian modified Delphi study. BMC Nursing, 15, 1–8. 10.1186/s12912-016-0166-2 27453690 PMC4957913

[nop2514-bib-0006] Burgess, A. , Ayton, T. , & Mellis, C. (2016). Implementation of team‐based learning in year 1 of a PBL based medical program: A pilot study. BMC Medical Education, 16, 49 10.1186/s12909-016-0550-3 26846425 PMC4743090

[nop2514-bib-0007] Celiker, H. D. (2015). Development of metacognitive skills: Designing problem‐based experiments with prospective science teachers in a biology laboratory. Educational Research and Reviews – Academic Journals, 10, 1487–1495. 10.5897/ERR2015.2283

[nop2514-bib-0008] Chiejina, E. N. , & Ebenebe, R. C. (2013). Metacognition: Usage by nursing students in the university. International Journal of Pure and Applied Sciences and Technology, 17(2), 1–8.ISSN2229‐6107.

[nop2514-bib-0009] Dinho, A. E. , & Swai, E. (2017). Tutor's clinical knowledge, attitude and teaching strategies in nursing schools of Mwanza Region – Tanzania. The International Annals of Medicine, 1(10), 1–6. 10.24087/IAM.2017.1.10.350

[nop2514-bib-0010] Dolamo, B. L. , & Olubiyi, S. K. (2013). Nursing education in Africa: South Africa, Nigeria and Ethiopia experiences. International Journal of Nursing and Midwifery, 5, 14–21. 10.5897/IJNM11.029

[nop2514-bib-0012] Gemuhay, H. M. , Kalolo, A. , Mirisho, R. , Chipwaza, B. , & Nyangena, E. (2019). Factors affecting performance in clinical practice among preservice diploma nursing students in northern Tanzania. Nursing Research and Practice, 2019, 1–9. 10.1155/2019/3453085 PMC6420979 30941212

[nop2514-bib-0013] Guilbert, J. J. (2006). The World Health Report 2006: Working together for health. Education for Health: Change in Learning and Practice, 19(3), 385–387. 10.1080/13576280600937911 17178522

[nop2514-bib-0014] Hamdan, A. R. , Kwan, C. L. , Khan, A. , Ghafar, M. N. A. , & Sihes, A. J. (2014). Implementation of problem‐based learning among nursing students. International Education Studies, 7, 136–142. 10.5539/ies.v7n7p136

[nop2514-bib-0015] Heid, C. L. (2016). Motivation and persistence among BSN students in Northeast Ohio: A correlational study. Motivation and persistence among BSN students in Northeast Ohio: A correlational study Escola Anna Nery, 20, 2, 243–247. http://www.gnresearch.org/doi/10.5935/1414‐8145.20160032

[nop2514-bib-0016] Hsu, L.‐L. , & Hsieh, S.‐I. (2014). Factors affecting the metacognition of undergraduate nursing students in a blended learning environment. International Journal of Nursing Practice, 20, 233–241. 10.1111/ijn.12131 24888995

[nop2514-bib-0017] Ismail, L.‐M.‐N. , Aboushady, R.‐M.‐N. , & Eswi, A. (2015). Clinical instructor's behavior: Nursing student's perception toward effective clinical instructor's characteristics. Journal of Nursing Education and Practice, 6(2), 96–105. http://www.sciedupress.com/journal/index.php/jnep/article/view/6941

[nop2514-bib-0018] Jamshidi, N. , Molazem, Z. , Sharif, F. , Torabizadeh, C. , & Kalyani, M. N. (2016). The challenges of nursing students in the clinical learning environment: A qualitative study. The Scientific World Journal, 2016, 1–7. 10.1155/2016/1846178 PMC4913003 27366787

[nop2514-bib-0019] Jost, M. , Brüstle, P. , Giesler, M. , Rijntjes, M. , & Brich, J. (2017). Effects of additional team‐based learning on students' clinical reasoning skills: A pilot study. BMC Research Notes, 10, 1–7. 10.1186/s13104-017-2614-9 28705246 PMC5512944

[nop2514-bib-0020] Kirschner, P. A. , Sweller, J. , & Clark, R. E. (2006). Why minimal guidance during instruction does not work: An analysis of the failure of constructivist, discovery, problem‐based, experiential and inquiry‐based teaching. Educational Psychologist, 41, 75–86. 10.1207/s15326985ep4102_1

[nop2514-bib-0021] Komba, S. C. , & Mwandanji, M. (2015). Reflections on the implementation of competence‐based curriculum in Tanzanian secondary schools. Journal of Education and Learning, 4(2), 73–80. 10.5539/jel.v4n2p73

[nop2514-bib-0022] Lawal, J. , Weaver, S. , Bryan, V. , & Lindo, J. L. (2015). Factors that influence the clinical learning experience of nursing students at a Caribbean school of nursing. Journal of Nursing Education and Practice, 6(4), 1–24. 10.5430/jnep.v6n4p32

[nop2514-bib-0023] Mafumiko, F. M. S. (2008). The Potential of Micro‐scale Chemistry Experimentation in enhancing teaching and learning of secondary chemistry: Experiences from Tanzania classrooms. NUE Journal of International Educational Cooperation, 3, 63–79.http://www.naruto‐u.ac.jp/_files/00107723/journal_03_09

[nop2514-bib-0024] Makunja, G. (2016). Challenges facing teachers in implementing competence‐based curriculum in Tanzania: The case of community secondary schools in Morogoro Municipality, Tanzania. International Journal of Education and Social Science, 3(5), 30–37. http//:www.ijessnet.com

[nop2514-bib-0025] Masek, A. , & Yamin, S. (2012). A comparative study of the effect of problem‐based learning and traditional learning approaches on students' knowledge acquisition BT – Current Trends in Nanotechnology Education. International Journal of Engineering Education, 28(5), 1161–1167. https://www.researchgate.net/publication/259873856

[nop2514-bib-0027] Melnyk, B. M. , Fineout‐Overholt, E. , Gallagher‐Ford, L. , & Kaplan, L. (2012). The state of evidence‐based practice in US nurses: Critical implications for nurse leaders and educators. Journal of Nursing Administration, 42, 410–417. 10.1097/NNA.0b013e3182664e0a 22922750

[nop2514-bib-0043] NACNEP, (2010). Addressing New Challenges Facing Nursing Education: Solutions for a Transforming Healthcare Environment. 1–25. http://www.hrsa.gov/advisoryhttp//:committees/bhpradvisory/nacnep/reports/eighthreport

[nop2514-bib-0028] Ojemeni, M. T. , Niles, P. , Mfaume, S. , Kapologwe, N. A. , Deng, L. , Stafford, R. , … Squires, A. (2017). A case study on building capacity to improve clinical mentoring and maternal child health in rural Tanzania: The path to implementation. BMC Nursing, 16, 1–9. 10.1186/s12912-017-0252-0 28959139 PMC5615632

[nop2514-bib-0042] Paslawski, T. K. , Ramona White, J. (2014). Measuring the Effectiveness of Faculty Facilitation Training in Problem‐Based Learning in a Medical School. Creative Education, 5(5), 164–170. 10.4236/ce.2014.54025\nhttp://creativecommons.org/licenses/by/4.0/

[nop2514-bib-0029] Paulo, A. (2014). Pre‐service teacher's preparedness to implement competence‐based curriculum in secondary schools in Tanzania. International Journal of Educational Research, 2(7), 2201–6333. http//:www.ijern.com

[nop2514-bib-0030] Marijani, R. (2017). Curriculation and Competence‐Based Education Training (CBET) in Tanzania: A Critical Assessment of Public Administration and Management (PAM) Curricula at Tanzania Public Service College (TPSC). HOLISTICA – Journal of Business and Public Administration, 8(2), 17–40. 10.1515/hjbpa-2017-0010

[nop2514-bib-0045] Shahin , Hanan, M. , Mohamed, T. & Eman Saleh, M. (2013). Critical thinking and self‐directed learning as an outcome of problem‐based learning among nursing students in Egypt and the Kingdom of Saudi Arabia. Journal of Nursing Education and Practice, 3(12), 103–110. 10.5430/jnep.v3n12p103

[nop2514-bib-0031] Souza, M. S. D. , Venkatesaperumal, R. , & Radhakrishnan, J. (2013). Engagement in the clinical learning environment among nursing students: Role of nurse educators. Open Journal of Nursing, 2013, 25–32. 10.4236/ojn.2013.31004

[nop2514-bib-0032] Stanley, M. J. C. , & Dougherty, J. P. (2010). A paradigm shift in nursing education: A new model. Nursing Education Perspectives, 31(6), 378–380.https://www.researchgate.net/publication/49797622 21280445

[nop2514-bib-0033] Tjoflåt, I. , & Våga, B. B. (2017). Implementing simulation in a nursing education program: A case report from Tanzania. BioMed Central, 2, 4–7. 10.1186/s41077-017-0048-z PMC5806360 29450018

[nop2514-bib-0034] Tsigarides, J. , Wingfield, L. R. , & Kulendran, M. (2017). Does a PBL – Based medical curriculum predispose training in specific career paths? A systematic review of the literature. BMC Research Notes, 10, 24 10.1186/s13104-016-2348-0 28061800 PMC5219658

[nop2514-bib-0035] Ulrich, B. , Early, S. , Krozek, C. , & Ashlock, C. H. (2010). Improving retention, confidence, and competence of new graduate nurses: Results from a 10‐Year Longitudinal Database. Nursing Economic, 28(6), 363–376. https://www.researchgate.net/publication/49807589 21291057

[nop2514-bib-0036] Wafaa, G. , & Nahed, A. (2010). Effect of problem‐based learning on nursing students' approaches to learning and their self‐directed learning abilities. International Journal of Academic Research., 2(4), 188–196. https://www.semanticscholar.org/paper/Medical‐students‐self‐efficacy‐in‐problem+STUDENTS+?+APPROACHES+TO+LEARNING+AND+THEIR+SELF+DIRECTED+LEARNING+ABILITIES#1

[nop2514-bib-0037] WHO (2013). Transforming and Scaling up health professionals' education and training World Health Organization Guideline 20123 (vol, 76), Geneva, Switzerland: WHO Library Cataloguing‐in‐Publication Data 10.1017/CBO9781107415324.004

[nop2514-bib-0038] WHO (2016). Nurse educator's core competencies Nursing Staff – education, WY 108, 20 Avenue Appia, 1211 Geneva 27, Switzerland: World Health Organization http://www.who.int

[nop2514-bib-0039] WHO (2017). Optimizing the contributions of the nursing and midwifery workforce to achieve universal health coverage and the sustainable development goals through education, research and practice Optimizing the contributions of the nursing and midwifery workforce to achieve universal health coverage and the sustainable development goals through education, research and practice, 12, Geneva, Switzerland: WHO Document Production Services Retrieved from: http://apps.who.int/iris

